# Identification of a New *de Novo* Mutation Underlying Regressive Episodic Ataxia Type I

**DOI:** 10.3389/fneur.2018.00587

**Published:** 2018-07-25

**Authors:** Zeynep S. Karalok, Alfredo Megaro, Marta Cenciarini, Alev Guven, Sonia M. Hasan, Birce D. Taskin, Paola Imbrici, Serdar Ceylaner, Mauro Pessia, Maria C. D'Adamo

**Affiliations:** ^1^Department of Pediatric Neurology, Ankara Children's Hematology Oncology Research and Training Hospital, Ankara, Turkey; ^2^Section of Physiology and Biochemistry, Department of Experimental Medicine, School of Medicine, University of Perugia, Perugia, Italy; ^3^Department of Physiology, Faculty of Medicine, Kuwait University, Kuwait, Kuwait; ^4^Department of Pharmacy-Drug Sciences, University of Bari Aldo Moro, Bari, Italy; ^5^Intergen Genetic Center, Ankara, Turkey; ^6^Department of Physiology and Biochemistry, Faculty of Medicine and Surgery, University of Malta, Msida, Malta

**Keywords:** episodic ataxia type 1(EA1), *KCNA1*, G311D, myokymia, diplopia

## Abstract

Episodic ataxia type 1 (EA1), a *Shaker*-like K^+^ *channelopathy*, is a consequence of genetic anomalies in the *KCNA1* gene that lead to dysfunctions in the voltage-gated K^+^ channel Kv1. 1. Generally, *KCNA1* mutations are inherited in an autosomal dominant manner. Here we report the clinical phenotype of an EA1 patient characterized by ataxia attacks that decrease in frequency with age, and eventually leading to therapy discontinuation. A new *de novo* mutation (c.932G>A) that changed a highly conserved glycine residue into an aspartate (p.G311D) was identified by using targeted next-generation sequencing. The conserved glycine is located in the S4–S5 linker, a crucial domain controlling Kv1.1 channel gating. *In silico* analyses predicted the mutation deleterious. Heterologous expression of the mutant (Kv1.1-G311D) channels resulted in remarkably decreased amplitudes of measured current, confirming the identified variant is pathogenic. Collectively, these findings corroborate the notion that EA1 also results from *de novo* variants and point out that regardless of the mutation-induced deleterious loss of Kv1.1 channel function the ataxia phenotype may improve spontaneously.

## Introduction

### Clinical description

A boy, 10 years of age, was admitted to Ankara Children's Hematology Oncology Research and Training Hospital complaining of recurrent cerebellar ataxia, abrupt onset of gait instability, and tremors of the chin, head (titubation), and hand. He was the first child born to a non-consanguineous couple with no medical history of note. Both, his sister and brother were healthy. The attacks started suddenly, lasted for 2 to 3 min during which he was conscious and able to answer questions appropriately. These attacks were video recorded by his parents and examined by a neurologist. Attacks occurred without precipitation, but were mostly triggered by fever. Home video recordings showed myokymia in the periorbital region and on the fingers of the patient. During attacks, the patient displayed diplopia without nystagmus. Symptoms such as nausea, tinnitus or headache were not reported by the patient. His developmental milestones and school performance were normal. Neurologic examinations performed between attacks were normal. Complete blood count, rate of erythrocyte sedimentation, blood urea nitrogen and thyroid function tests, vitamin A and E levels, lipid electrophoresis tests, electrolyte, glucose, and magnesium levels were also normal. Heart rate, ECG parameters, cranial magnetic resonance imaging (MRI), and electroencephalography (EEG) records were normal, as well as sleep habits and body temperature. Electromyography (EMG) investigations showed normal nerve conduction velocities for both motor and sensory nerves, as well as normal distal motor latencies. The presence of myokymia was established by the analysis of videos recorded during the attacks that initially occurred 4 to 5 times a year, but then the frequency decreased to 2 to 3 times a year. Due to slowly improving symptoms, the proband's parents discontinued the treatment with acetazolamide, oxcarbazepine, and valproic acid after 6–9 months of ataxia free period.

### Mutation analysis

**Figure 1:**
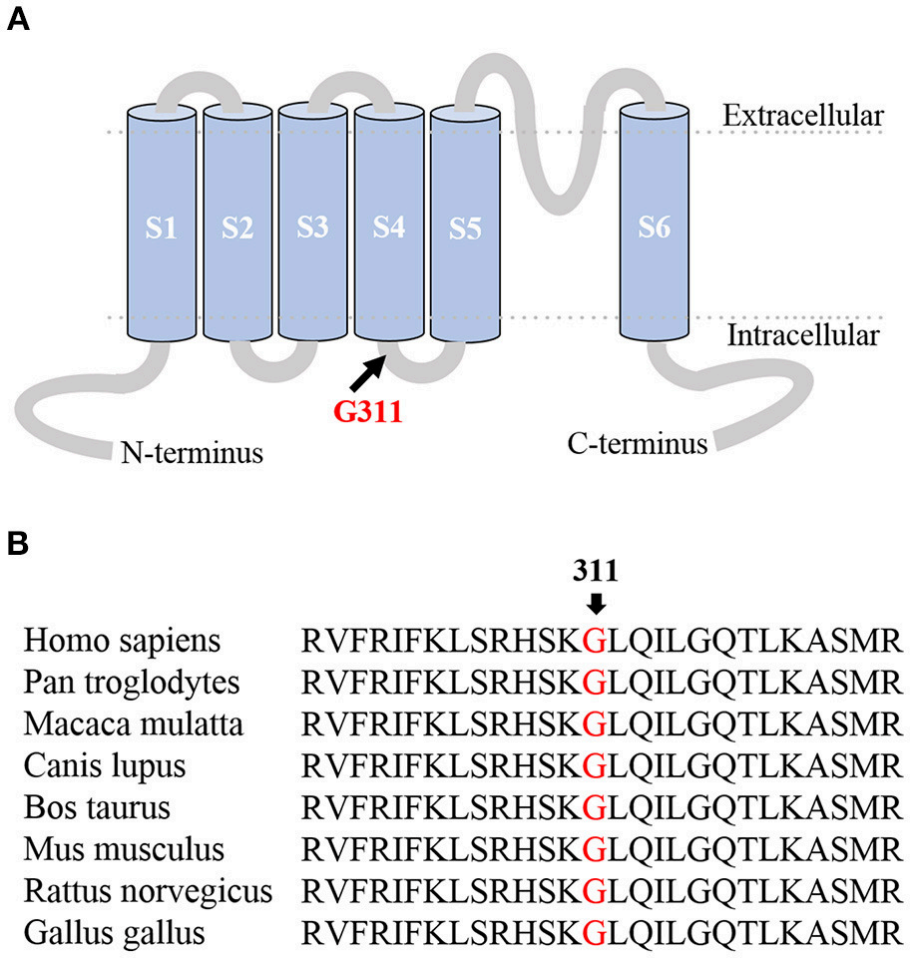
**(A)** Membrane topology model of the Kv1.1 α subunit. The G311D mutation occurred at a highly conserved residue located in the S4-S5 linker domain. **(B)** Protein multisequence alignment of the Kv1.1 region of interest. The alignment, obtained by using MUSCLE 3.6, showed the Glycine at position 311 is highly conserved among species.

The proband's clinical phenotype was typical of EA1. Genetic testing was performed by means of targeted next-generation sequencing (NGS). Exons 1 to 2 of *KCNA1*, the only gene associated with EA1, and their flanking splice site junctions were amplified, sequenced, and compared with the reference sequence deposited in the NCBI database (NM_000217.2). This analysis showed the presence of a missense mutation in exon 2 of the *KCNA1* gene (c.932G>A) carried in a heterozygous state. Multisequence alignment showed that the resulting p.G311D mutation occurred at a highly conserved residue (Figure 1B) located in the S4–S5 linker domain (Figure 1A). NGS of exons 1 to 2 of the *KCNA1* gene and their flanking splice site junctions was performed for both parents, resulting in normal sequences. Three different *in silico* analyses were then performed in order to evaluate the pathogenic relevance of the identified mutation. Consistently, SIFT (Sorting Intolerant from Tolerant program; http://sift.jcvi.org/), MT (Mutation Taster program; http://www.mutationtaster.org), and PolyPhen2 (http://genetics.bwh.harvard.edu/pph2/) softwares predicted the mutation was deleterious.

### Background

Amongst the first discovered *K*^+^ *channelopathies*, EA1 [OMIM 160120] became an early member of a long list of human diseases caused by ion channel dysfunction (1–7). Despite new mutations being detected, the actual prevalence of EA1 is uncertain and may be considerably higher than estimates most likely due to incorrect diagnosis. EA1 is an autosomal dominant disorder associated with *KCNA1* that codes the voltage-dependent K^+^ channel Kv1.1. Indeed, several heterozygous missense mutations have been identified that resulted in different expression and gating defects in the Kv1.1 channel (1, 8–16). The disease is distinguished by symptoms that include muscle twitching (myokymia) and an episodic loss of motor control, coordination, and balance that is associated with spastic head, arm, and leg muscle contractions. Episode intervals vary from patient to patient, but are predominantly short (seconds to minutes) with occasional cases of prolonged attacks that last hours and days (17). Patients may, in the course of an episode, experience vertigo, distorted or double vision, queasiness, headache, sweating, muscle stiffening, hyperthermia, and dysarthria. Symptoms appear initially during childhood, sometimes as a consequence of physical or emotional trauma (18, 19). An episode may be brought on by repetition of knee bend exercises or vestibular caloric stimulation. In addition, fever, fatigue, abrupt movements or a startle, anxiety, and caffeine are possible triggers. On the other hand, myokymia occurs continuously, both within and between episodes, and is visible as fine flowing movement of the perioral or periorbital muscles and/or as involuntary quivering of the fingers. EA1 cases without myokymia have been reported, however regional ischemia may make the myokymia apparent (17, 19). Investigations performed on patients or rodents with EA1 mutations showed changes in axonal excitability (20, 21).

Here we report the identification of a novel *de novo* mutation associated with a slowly ameliorating EA1 phenotype.

### Functional characterization of the mutated channel

**Figure 2:**
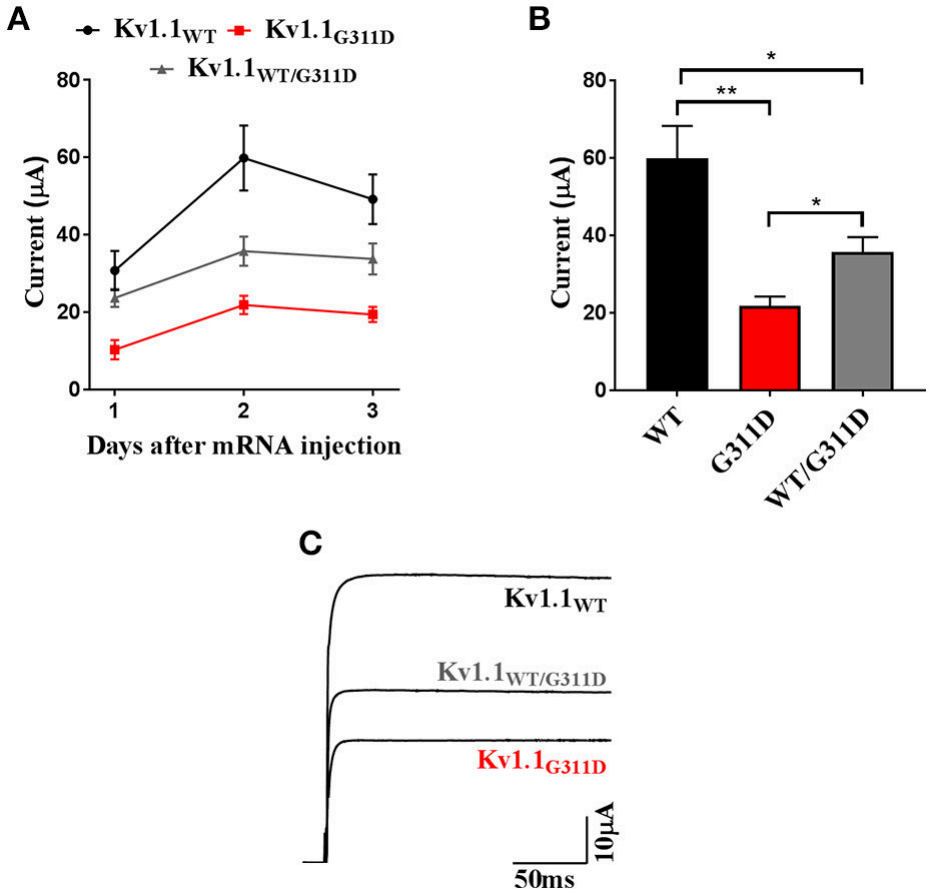
Heterologous expression of Kv1.1-WT and G311D mutant channels in Xenopus oocytes. **(A)** Averaged current amplitudes computed at +60 mV for Kv1.1-WT, G311D, and WT+G311D channels at 24, 48, and 72 h post cRNA injections. Each data point is the mean±SE of 8 cells.**(B)** Bar graph current amplitudes recorded 48 h after injection. The mutant channel shows ~3-fold smaller current than the WT (*p < 0.05, **p < 0.005, Student *t*-test). **(C)** Representative Kv1.1 current traces recorded at +60 mV from a holding potential of−80 mV showing reduction in oocytes expressing G311D cRNA.

To validate the pathogenic relevance of the identified variant *in vitro*, Kv1.1-WT, and Kv1.1-G311D cRNAs were heterologously expressed in Xenopus oocytes (methods are described in the Supplementary Material). Current families were recorded in TEVC configuration and induced by depolarizing steps from a holding potential of −80 mV to a range of −70 to +60 mV test potentials (each step duration was 200 ms). The expression of both channel types resulted in functional delayed-rectifying K^+^ currents. Nevertheless, the average current amplitude computed at +60 mV for the mutant channel was ~3-fold smaller than the WT (Figure 2). The G311D mutation was carried by the proband in a heterozygous state. Assuming that the normal and mutant alleles are both expressed, WT and mutant subunits may form heteromeric channels in the plasma membrane of nerve cells. Co-expression experiments were performed whereby WT and mutant RNAs in a 1:1 ratio were co-injected in the same oocyte. At +60 mV, the co-injected oocytes had an ~50% decrease in mean current amplitude when compared to the mean amplitude measured from oocytes injected with control WT cRNA (Figure 2).

## Discussion

Despite the wide phenotypic spectrum reported in EA1 and the progress in characterizing its mutations (17), this is the first report since the discovery of EA1 in 1975 (22) that clearly describes a patient showing attack frequency decrease with age along with amelioration of EA1 symptoms. This slowly regressing EA1 phenotype eventually led to therapy discontinuation.

Surprisingly, despite the strong pathogenicity score of the mutation, our proband had mild symptoms. Some affected EA1 patients displayed severe cognitive dysfunctions with language delay, both receptive and expressive. Some have difficulty learning tasks that require co-ordination such as riding a bicycle. Some EA1 children require special coping programs and schools that deal with learning difficulties. However, the developmental milestones and school performance of our proband were normal. Neuromuscular findings observed in EA1 patients include unusual hypercontracted posture, contractures (elbow, hip, and knee), abdominal wall muscle contraction, and shortened calcaneal tendons that lead to toe walking. Cases with skeletal deformities such as scoliosis, kyphoscoliosis, high arched palate, and minor craniofacial dysmorphism have been reported. Nevertheless, our proband did not display any of these features. During the attacks patients experience gait changes; only ~25% of patients can walk independently, 50% of patients need help, and nearly 20% of patients are unable to walk (23). The patient described here did not require much assistance in walking. MRI evaluation of the proband's brain and spinal cord resulted in values within the normal range. Literature review showed the brain MRI of 11 out of 12 (91.7%) EA1 patients to be normal. Since only one patient had cerebellar atrophy, a relationship between this neuroanatomical abnormality and EA1 has not been determined (23). EA1 patients are 10 times more prone to acquire epilepsy (24). Our patient did not have seizure-like symptoms and his EEG recordings were normal. Motor and sensory nerve conduction and functions were normal compared to healthy subjects. Yet despite the milder phenotype, our proband did initially experience typical episodic signs of EA1.

A heterozygous mutation changing the glycine residue at position 311 into a serine has been previously reported (25). Regrettably, the clinical phenotype of the patient carrying this mutation and the inheritance mode was not described making genotype-phenotype comparisons and correlations unfeasible. Zerr et al. showed the homomeric G311S mutation produced a reduction in surface expression and function of the channel similar to that of G311D. In our study, the functional characterization of both homomeric and heteromeric G311D channels expressed in oocytes showed that potassium currents are markedly reduced. This reduction may be a result of *haploinsufficiency*. However, changes in channel gating and kinetics may also be the reason behind current decrease since the mutation is located in the S4-S5 linker, a crucial domain controlling Kv1.1 channel gating (Figure 1B). The substitution of glycine, a neutral nonpolar residue that prefers a hydrophobic environment, with an acidic polar residue that energetically favors contact with water more than likely resulted in changes in the channel's conformation and in the bonds between the 311 residue and surrounding amino acids. Mutations that weaken or eliminate hydrophobic interactions between residues have been shown to affect the kinetics and voltage-dependence of gating, and to manifest as the EA1 phenotype in patients (26). Future investigations will include determining the important role this highly conserved glycine plays at this position by assessing the effect of the G311D mutation on the channel's biophysical parameters.

Kv1.1-containing channels regulate a number of crucial functions that include the outflow of K^+^, resting membrane potential, neuronal excitability, action potential threshold, waveforms, and frequency, and the release of neurotransmitters at axon terminals (27–29). The G311D-induced haploinsufficiency may alter some of these functions, resulting in EA1 symptoms. Loss of channel function results in the lengthening of action potential duration of the neurons that express Kv1.1, such as in the Kv1.1-abundant basket cell terminals. The resulting enhancement of Ca^2+^ transients may lead to a greater γ-aminobutyric acid (GABA) discharge from basket cell presynaptic terminals, thereby reducing Purkinje cell inhibitory output. As a result, deep cerebellar nuclei would be hyper-excitable, altering the entire cerebellar output to the brain and ultimately causing the distinctive EA1 cerebellar symptoms [*see* (9, 16)]. Nevertheless, the exact mechanisms by which Kv1.1 mutations alter nerve cell properties leading to the overt EA1 phenotype and to the episodicity of the attacks still awaits clarification.

Although EA1 patients predominantly inherit their mutation from an affected parent, *de novo* mutations have been found (30, 31). Here we report a novel variant that further corroborates the notion that EA1 also stems from *de novo* germline mutations. Therefore, for correct EA1 diagnosis molecular genetic testing of *KCNA1* is recommended even in the absence of family history. It is still possible, however, that other genes may contribute or be responsible for EA1, since cases have been reported with characteristic EA1 symptoms without *KCNA1* variants being present.

## Concluding remarks

In this study, we report a new *de novo* mutation and an accompanying regressive EA1 phenotype indicating that compensatory mechanisms that circumvent the deleterious effects of mutations may take place in the brain. Future challenges should be focused on uncovering these mechanisms in order to identify innovative therapies for EA1 and other channelopathies.

## Ethics statement

Studies involving the proband and his family complied with the Helsinki Declaration and was approved by the Ethical Committee of University of Health Sciences, Ankara Children Health, and Diseases Hematology-Oncology Training and Research Hospital Clinical Research Ethics Committee. Written Informed consent for participation and publishing were obtained from the proband's parents on behalf of the proband (aged < 18 years).

## Author contributions

ZK designed the work and wrote the manuscript. AM performed electrophysiological experiments. MC performed the molecular biology experiments. AG performed data analysis. SH reviewed statistics, provided figures and edited the manuscript. BT planned the genetic analysis and interpreted the results. PI critically discussed the manuscript. SC performed the genetic screening. MP critically discussed the results and supervised the work. MD designed and supervised the work, reviewed the literature, and wrote the manuscript.

### Conflict of interest statement

The authors declare that the research was conducted in the absence of any commercial or financial relationships that could be construed as a potential conflict of interest.

## References

[1] Adelman J Bond CT Pessia M Maylie J. Episodic ataxia results from voltage-dependent potassium channels with altered functions. Neuron (1995) 15:1449–54. 10.1016/0896-6273(95)90022-5 8845167

[2] Sicca F Imbrici P D'Adamo MC Moro F Bonatti F Brovedani P. Autism with seizures and intellectual disability: possible causative role of gain-of-function of the inwardly-rectifying K^+^ channel Kir4.1. Neurobiol Dis. (2011) 43:239–47. 10.1016/j.nbd.2011.03.016 21458570

[3] D'Adamo MC Catacuzzeno L Di Giovanni G Franciolini F Pessia M. K^+^ channelepsy: progress in the neurobiology of potassium channels and epilepsy. Front Cell Neurosci. (2013) 7:134. 10.3389/fncel.2013.00134 24062639 PMC3772396

[4] Ambrosini E Sicca F Brignone MS D'Adamo MC Napolitano C Servettini I. Genetically induced dysfunctions of Kir2.1 channels: implications for short QT_3_ syndrome and autism-epilepsy phenotype. Hum Mol Genet. (2014) 23:4875–86. 10.1093/hmg/ddu201 24794859 PMC4140467

[5] Guglielmi L Servettini I Caramia M Catacuzzeno L Franciolini F D'Adamo MC. Update on the implication of potassium channels in autism: K^+^ channel autism spectrum disorder. Front Cell Neurosci. (2015) 9:34. 10.3389/fncel.2015.00034 25784856 PMC4345917

[6] Parolin Schnekenberg R Perkins EM Miller JW Davies WI D'Adamo MC Pessia M. De novo point mutations in patients diagnosed with ataxic cerebral palsy. Brain (2015) 138:1817–32. 10.1093/brain/awv117 25981959 PMC4572487

[7] Hasan S Balobaid A Grottesi A Dabbagh O Cenciarini M Rawashdeh R Lethal digenic mutations in the K+ channels Kir4.1 (KCNJ10) and SLACK (KCNT1) associated with severe-disabling seizures and neurodevelopmental delay. J Neurophysiol. (2017) 4:2402–11. 10.1152/jn.00284.2017 PMC5646198 28747464

[8] D'Adamo MC Liu Z Adelman JP Maylie J Pessia M. Episodic ataxia type-1 mutations in the hKv1.1 cytoplasmic pore region alter the gating properties of the channel. EMBO J. (1998) 17:1200–7. 10.1093/emboj/17.5.1200 9482717 PMC1170468

[9] D'Adamo MC Imbrici P Sponcichetti F Pessia M. Mutations in the *KCNA*1 gene associated with episodic ataxia type-1 syndrome impair heteromeric voltage-gated K^+^ channel function. FASEB J. (1999) 13:1335–45. 10.1096/fasebj.13.11.1335 10428758

[10] Imbrici P Cusimano A D'Adamo MC De Curtis A Pessia M. Functional characterization of an episodic ataxia type-1 mutation occurring in the S1 segment of hKv1.1 channels. Pflugers Arch. (2003) 446:373–9. 10.1007/s00424-002-0962-2 12799903

[11] Imbrici P D'Adamo MC Kullmann DM Pessia M. Episodic ataxia type 1 mutations in the *KCNA1* gene impair the fast inactivation properties of the human potassium channels Kv1.4-1.1/Kvbeta1.1 and Kv1.4-1.1/Kvbeta1.2. Eur J Neurosci. (2006) 24:3073–83. 10.1111/j.1460-9568.2006.05186.x 17156368

[12] Imbrici P D'Adamo MC Cusimano A Pessia M. Episodic ataxia type 1 mutation F184C alters Zn^2+^-induced modulation of the human K^+^ channel Kv1.4-Kv1.1/Kvbeta1.1. Am J Physiol Cell Physiol. (2007) 292:C778–87. 10.1152/ajpcell.00259.2006 16956965

[13] Imbrici P Grottesi A D'Adamo MC Pessia M. Contribution of the central hydrophobic residue in the *PXP* motif of voltage-dependent K^+^ channels to S6 flexibility and gating properties. Channels (2009) 3:39–45. 19202350 10.4161/chan.3.1.7548

[14] Imbrici P D'Adamo MC Grottesi A Biscarini A Pessia M. Episodic ataxia type 1 mutations affect fast inactivation of K^+^ channels by a reduction in either subunit surface expression or affinity for inactivation domain. Am J Physiol Cell Physiol. (2011) 300:C1314–22. 10.1152/ajpcell.00456.2010 21307345

[15] Cusimano A D'Adamo MC Pessia M. An episodic ataxia type-1 mutation in the S1 segment sensitises the hKv1.1 potassium channel to extracellular Zn^2+^. FEBS Lett. (2004) 576:237–44. 10.1016/j.febslet.2004.09.018 15474044

[16] Hasan S Bove C Silvestri G Mantuano E Modoni A Veneziano L. A channelopathy mutation in the voltage-sensor discloses contributions of a conserved phenylalanine to gating properties of Kv1.1 channels and ataxia. Sci Rep. (2017) 7:4583. 10.1038/s41598-017-03041-z 28676720 PMC5496848

[17] D'Adamo MC Hasan S Guglielmi L Servettini I Cenciarini M Catacuzzeno L. New insights into the pathogenesis and therapeutics of episodic ataxia type 1. Front Cell Neurosci. (2015) 9:317. 10.3389/fncel.2015.00317 26347608 PMC4541215

[18] Imbrici P Gualandi F D'Adamo MC Masieri MT Cudia P De Grandis D. A novel *KCNA1* mutation identified in an Italian family affected by episodic ataxia type 1. Neuroscience (2008) 157:577–87. 10.1016/j.neuroscience.2008.09.022 18926884

[19] Hasan SM D'Adamo MC. Episodic ataxia type 1. In: Adam MP Ardinger HH Pagon RA., editors. GeneReviews® [Internet]. Seattle, WA: University of Washington, Seattle (2018).

[20] Brunetti O Imbrici P Botti FM Pettorossi VE D'Adamo MC Valentino M. Kv1.1 knock-in ataxic mice exhibit spontaneous myokymic activity exacerbated by fatigue, ischemia and low temperature. Neurobiol Dis. (2012) 47:310–21. 10.1016/j.nbd.2012.05.002 22609489 PMC3402927

[21] Tomlinson SE Tan SV Kullmann DM Griggs RC Burke D Hanna MG. Nerve excitability studies characterize Kv1.1 fast potassium channel dysfunction in patients with episodic ataxia type 1. Brain (2010) 133:3530–40. 10.1093/brain/awq318 21106501 PMC2995887

[22] Van Dyke DH Griggs RC Murphy MJ Goldstein MN Hereditary myo-kymia and periodic ataxia. J Neurol Sci. (1975) 25:109–18. 10.1016/0022-510X(75)90191-4 1170284

[23] Graves TD Cha YH Hahn AF Salajegheh MK Griggs RC Bundy BN. Episodic ataxia type 1: clinical characterization, quality of life and genotype-phenotype correlation. Brain (2014) 137:1009–18. 10.1093/brain/awu012 24578548 PMC3959554

[24] Zuberi SM Eunson LH Spauschus A De Silva R Tolmie J Wood NW. A novel mutation in the human voltage-gated potassium channel gene (Kv1.1) associates with episodic ataxia type 1 and sometimes with partial epilepsy. Brain (1999) 122:817–25. 10.1093/brain/122.5.817 10355668

[25] Zerr P Adelman JP Maylie J. Characterization of three episodic ataxia mutations in the human Kv1.1 potassium channel. FEBS Lett. (1998) 431:461–4. 10.1016/S0014-5793(98)00814-X 9714564

[26] Hasan S Hunter T Hunter G Pessia M D'Adamo MC. Commentary: A channelopathy mutation in the voltage-sensor discloses contributions of a conserved phenylalanine to gating properties of Kv1.1 channels and ataxia. Front. Cell. Neurosci. (2018) 12:174. 10.3389/fncel.2018.00174 29973872 PMC6019458

[27] Kuba H Yamada R Ishiguro G Adachi R. Redistribution of Kv1 and Kv7 enhances neuronal excitability during structural axon initial segment plasticity. Nat Commun. (2015) 6:8815. 10.1038/ncomms9815 26581625 PMC4673506

[28] Johnston J Forsythe ID Kopp-Scheinpflug C. Going native: voltage-gated potassium channels controlling neuronal excitability. J Physiol. (2010) 588:3187–200. 10.1113/jphysiol.2010.191973 20519310 PMC2976014

[29] Begum R Bakiri Y Volynski KE Kullmann DM. Action potential broadening in a presynaptic channelopathy. Nat Commun. (2016) 7:12102. 10.1038/ncomms12102 27381274 PMC4935806

[30] Demos MK Macri V Farrell K Nelson TN Chapman K Accili E. A novel *KCNA1* mutation associated with global delay and persistent cerebellar dysfunction. Mov Disord. (2009) 24:778–82. 10.1002/mds.22467 19205071

[31] Lassche S Lainez S Bloem BR van de Warrenburg BP Hofmeijer J Lemmink HH. A novel *KCNA1* mutation causing episodic ataxia type I. Muscle Nerve. (2014) 50:289–91. 10.1002/mus.24242 24639406

