# Supplementary material for: Identification of a New de Novo Mutation Underlying Regressive Episodic Ataxia Type I
Source: Front Neurol. 2018 Jul 25;9:587. doi: 10.3389/fneur.2018.00587 (PMC6094999; doi:10.3389/fneur.2018.00587)
Supplement: Supplementary file 1 [file Data_Sheet_1.DOCX]

**Supplementary materials**

Methods

**Clinical and neurophysiologic examination**

Studies involving the proband and his family complied with the Helsinki Declaration and was approved by the Ethical Committee of University of Health Sciences, Ankara Children Health and Diseases Hematology-Oncology Training and Research Hospital Clinical Research Ethics Committee. Written Informed consent for participation and publishing were obtained from the proband’s parents on behalf of the proband (aged <18 years).

The neurophysiological evaluation included motor and sensory nerve conduction studies. A needle EMG study could not be performed. Patient was examined in a comfortable room with skin temperature maintained above 32°C. Motor nerve conduction studies were carried out on median, ulnar, posterior tibialis, and common peroneal nerves. Sensory nerve conduction studies were carried out on median, ulnar, and sural nerves. Cranial magnetic resonance imaging (MRI) and electroencephalography (EEG) were performed and analysed by a paediatric radiologist.

Genetic analysis

DNA was isolated from peripheral blood using standard methods. Exons 1 to 2 of the *KCNA1* gene and their flanking splice site junctions were amplified using primers and protocols from PRIMER – Primer Designer v.2.0 (Scientific & Educational Software program). Next-generation sequencing (NGS) of the *KCNA1* gene was performed by using the MiSeq NGS platform (Illumina, San Diego, CA, USA). Three different *in silico* analyses were performed. Sorting Intolerant From Tolerant program (SIFT; <http://sift.jcvi.org/>), Mutation Taster program ([http://www.mutationtaster.org](http://www.mutationtaster.org/)), and PolyPhen2 program (<http://genetics.bwh.harvard.edu/pph2/>) were used to predict the pathogenicity of the variant found. DANN 0.9978 Mutation Taster 1, Allele not found in Broad gnomAD exomes, Missense variant in gene KCNA1 which has 30 pathogenic variants vs 1 benign missense variant.

**Mutagenesis**

Human Kv1.1 cDNA was subcloned into a pBF oocyte expression vector. The mutation p.G311D was introduced by site-directed mutagenesis performed using QuickChange protocol (Stratagene, La Jolla, CA, USA) and was veriﬁed by automated sequencing. The cRNAs of Kv1.1WT and G311D were transcribed *in vitro* by using mMESSAGE mMACHINE™ SP6 Transcription Kit (Ambion, Life technologies, Carlsbad, CA, USA) and concentrations were quantiﬁed by electrophoresis with ethidium bromide staining and spectrophotometric analysis.

**Expression of *KCNA1* constructs in *Xenopus laevis* oocytes**

Wild-type and mutant channels were expressed in *Xenopus laevis* oocytes as described in Hasan et al., 2017. Animal handling was in accordance with international standards of animal care, the Maltese Animal Welfare Act approved by the local Veterinary Service Authority, and the NIH Guide for the Care and Use of Laboratory Animals. *Xenopus laevis* were deeply anesthetized with an aerated solution containing 3-aminobenzoic acid ethyl ester methanesulfonate salt (5mM) and sodium bicarbonate (60mM), pH 7.3. Stage V–VI *Xenopus laevis* oocytes were isolated, digested with Collagenase Type A, injected with cRNA (50nl each) and incubated at 16^◦^C in ND96 solution (pH 7.4) containing in mM: NaCl 96, KCl 2, MgCl_2_ 1, CaCl_2_ 1.8, HEPES 5, and containing gentamicin 50μg/ml. All chemicals were purchased by Sigma-Aldrich.

**Electrophysiology**

Two-electrode voltage-clamp (TEVC) recordings were performed as previously described (Hasan et al., 2017). Briefly, whole-cell currents were recorded using TEVC on oocytes at ~22^◦^C. Recordings were made 24, 48 and 72 hrs after cRNA injection, by using a GeneClamp 500 amplifier (Axon Instruments, Foster City, CA) interfaced to a PC computer with an ITC-16 interface (InstruTech, Port Washington, NY). Microelectrodes were pulled to a tip resistance of <1MΩ. The extracellular solution contained (mM): NaCl 96, KCl 2, MgCl_2_ 1, CaCl_2_ 1.8, HEPES 5, pH7.4. Recordings were analyzed with either PulseFit (HEKA, Germany) or Origin 8 (OriginLab, Northampton, MA). Leak and capacitive currents were subtracted using a P/4 protocol.

**Statistical Analysis**

Statistical analysis was performed using the software program Prism 7.04 (GraphPad Software, San Diego, CA). All data are shown as mean ± standard error (SE). Observed differences were evaluated by two-tailed unpaired Student’s t-test and were considered significant if p<0.05.
